# Radiomic analysis of lung cancer for the assessment of patient prognosis and intratumor heterogeneity

**DOI:** 10.1590/0100-3984.2019.0135

**Published:** 2021

**Authors:** José Raniery Ferreira Junior, Marcel Koenigkam-Santos, Camila Vilas Boas Machado, Matheus Calil Faleiros, Natália Santana Chiari Correia, Federico Enrique Garcia Cipriano, Alexandre Todorovic Fabro, Paulo Mazzoncini de Azevedo-Marques

**Affiliations:** 1 Faculdade de Medicina de Ribeirão Preto da Universidade de São Paulo (FMRP-USP), Ribeirão Preto, SP, Brazil.

**Keywords:** Tomography, X-ray computed, Radiographic image interpretation, computer-assisted, Lung neoplasms, Prognosis, Tomografia computadorizada, Interpretação de imagem radiográfica assistida por computador, Neoplasia pulmonar, Prognóstico

## Abstract

**Objective:**

To determine whether the radiomic features of lung lesions on computed tomography correlate with overall survival in lung cancer patients.

**Materials and Methods:**

This was a retrospective study involving 101 consecutive patients with malignant neoplasms confirmed by biopsy or surgery. On computed tomography images, the lesions were submitted to semi-automated segmentation and were characterized on the basis of 2,465 radiomic variables. The prognostic assessment was based on Kaplan-Meier analysis and log-rank tests, according to the median value of the radiomic variables.

**Results:**

Of the 101 patients evaluated, 28 died (16 dying from lung cancer), and 73 were censored, with a mean overall survival time of 1,819.4 days (95% confidence interval [95% CI]: 1,481.2-2,157.5). One radiomic feature (the mean of the Fourier transform) presented a difference on Kaplan-Meier curves (*p* < 0.05). A high-risk group of patients was identified on the basis of high values for the mean of the Fourier transform. In that group, the mean survival time was 1,465.4 days (95% CI: 985.2-1,945.6), with a hazard ratio of 2.12 (95% CI: 1.01-4.48). We also identified a low-risk group, in which the mean of the Fourier transform was low (mean survival time of 2,164.8 days; 95% CI: 1,745.4-2,584.1).

**Conclusion:**

A radiomic signature based on the Fourier transform correlates with overall survival, representing a prognostic biomarker for risk stratification in patients with lung cancer.

## Abstract (pt)

**Objetivo:**

Associar características radiômicas de lesões pulmonares em imagens de tomografia computadorizada com a sobrevida global de pacientes com câncer de pulmão.

**Materiais e Métodos:**

Estudo retrospectivo composto por 101 pacientes consecutivos com neoplasia maligna confirmada por biópsia/cirurgia. As lesões foram semiautomaticamente segmentadas e caracterizadas por 2.465 variáveis radiômicas. A avaliação prognóstica foi baseada na análise de Kaplan-Meier e no teste *log-rank*, de acordo com a mediana dos valores das variáveis.

**Resultados:**

Vinte e oito pacientes faleceram (16 por câncer de pulmão) e 73 foram censurados, com tempo médio de sobrevida de 1.819,4 dias (intervalo de confiança 95% [IC 95%]: 1.481,2-2.157,5). Uma característica radiômica (média de Fourier) apresentou diferença nas curvas de Kaplan-Meier (*p* < 0,05). Um grupo de pacientes de maior risco foi identificado a partir de valores altos da variável: sobrevida de 1.465,4 dias (IC 95%: 985,2-1.945,6) e razão de risco de 2,12 (IC 95%: 1,01-4,48). Um grupo de menor risco foi identificado a partir de valores baixos da variável (sobrevida de 2.164,8 dias; IC 95%: 1.745,4-2.584,1).

**Conclusão:**

Este estudo apresentou uma assinatura radiômica em imagens de tomografia computadorizada, baseada na transformada de Fourier, correlacionada com a sobrevida global de pacientes com câncer de pulmão, representando assim um biomarcador prognóstico.

## INTRODUCTION

Lung cancer is the leading cause of cancer-related death worldwide, accounting for one in five deaths^(1)^. Defining the prognosis of lung cancer is a major challenge, because it can vary dramatically depending on the tumor stage at diagnosis^(2)^. The choice of treatment for a malignant tumor is made primarily on the basis of the international neoplasm staging system, also known as the tumor-node-metastasis (TNM) system^(3)^. However, studies have shown that other clinical factors, such as tumor histological type and the presence of specific genetic mutations, can also affect the prognosis, the clinical decision-making process, and the treatment^(4,5)^.

Computed tomography (CT) is the imaging method most widely used for diagnosing and staging lung cancer^(6)^. In routine clinical practice, lung cancer is assessed on the basis of tumor size, signs of invasion into adjacent structures, lymph node involvement, and lesions suggestive of distant metastases. However, as other studies have shown^(2,7)^, in addition to the TNM staging, other tumor imaging features can also affect prognosis and the therapeutic decision-making process. Those imaging features usually relate to the shape and attenuation pattern of the lesions (such as heterogeneous enhancement, enhancement intensity, spiculated contours, and two-dimensional diameter on the axial plane) and are evaluated in a subjective, qualitative, or semi-quantitative manner^(8,9)^.

Radiomics, on the other hand, has been described as a promising, quantitative, reproducible tool for the characterization of medical images^(10,11)^. Simply put, radiomics performs a massive extraction of quantitative imaging variables and then a computer analysis of those variables, combining them with clinical and biochemical data related not only to diagnosis, but also to clinical outcome, histological data, and genetic mutations, increasing the power of biomarkers and decision support systems^(12-14)^. Radiomics is also able to quantify the spatial complexity of tumors and to identify tumor heterogeneity, which is the presence of multiple histological and genetic subregions within a tumor, a feature that can be related to disease progression and treatment resistance^(15,16)^. In view of recent advances in targeted therapies and immunotherapies, it is now imperative to carry out comprehensive and individualized assessments of neoplasms, and radiomics can do this in a noninvasive, rapid, low-cost manner in routine clinical practice^(17,18)^.

The objective of the present study was to determine whether the radiomic analysis of lung cancer lesions on CT images correlates with prognosis and overall survival in patients with lung cancer.

## MATERIALS AND METHODS

### Patients

This was a retrospective study. The study was approved by the research ethics committee of our institution. Because of the retrospective nature of the study, the requirement for written informed consent was waived. The initial sample included 126 consecutive patients with lesions consistent with lung cancer, mainly pulmonary nodules, confirmed by histology or surgery, who were referred for further investigation and diagnosis after a multidisciplinary discussion. Patients were diagnosed and treated between 2010 and 2017 at one of the hospitals operated by our institution. Of those 126 cases, 25 were excluded from the analysis: 19 because the standard CT protocol for administration of intravenous iodinated contrast medium was not followed (which affected the image characterization process) or because the images showed significant artifacts; four because there were other opacities adjacent to the tumor (which affected the segmentation process); and two because not all of the clinical data were available. The 101 cases included had diagnostic-quality contrast-enhanced CT images and all the necessary clinical data available for the analysis. The clinical and pathological data were obtained from the electronic medical records of the patients (Table 1).

**Table 1:** Main clinical, pathological, and imaging features of the malignant lung lesions in our sample.

Feature	N = 101
Age (years), mean ± SD (range)	66.4 ± 9.4 (41-85)
Gender (female/male), n	45/56
Smoking history (yes/no), n	84/17
History of another primary cancer (yes/no), n	33/68
Histopathology (ADC/SCC/other), n	58/27/16
T stage (1/2/3/4), n	39/47/6/9
N stage (0/1/2/3), n	53/20/20/8
M stage (0/1), n	79/22
Location (RUL/ML/RLL/LUL/LLL), n	31/3/27/20/20
Position (central/peripheral), n	6/95
Diameter (millimeters), mean ± SD (range)	32.3 ± 14.6 (10-98)

ADC, adenocarcinoma; SCC, squamous cell carcinoma; other, 7 small cell carcinomas, 5 carcinoids, 1 poorly differentiated carcinoma, 1 unspecified carcinoma, 1 large call carcinoma, 1 adenosquamous carcinoma; RUL, right upper lobe; ML, middle lobe; RLL, right lower lobe; LUL, left upper lobe; LLL, left lower lobe.

### CT image acquisition

Before any diagnostic or therapeutic intervention, the patients underwent CT in a 16-slice scanner (Brilliance Big Bore; Philips Healthcare, Eindhoven, Netherlands) or in a 128-slice scanner (Aquilion Prime; Toshiba Medical Systems, Tokyo, Japan). In all examinations, the image acquisition and reconstruction protocols were similar, varying depending on the clinical routine of the institution. The chest examinations were performed during a deep inspiratory breath hold, in a single volumetric acquisition in the caudocranial direction, with automatic exposure control, after intravenous administration of 80-100 mL of iodinated contrast medium (flow rate, 3.0 mL/s) adjusted according to patient weight in a rapid bolus injection, followed by injection of 30 mL of saline solution (flow rate, 3.0 mL/s). The images were reconstructed using a 512 × 512 matrix, a slice thickness of 0.5-1.25 mm, a standard filter (for the radiomic analysis), and a hard filter (used for visualization and manual measurement of the lesions in lung window settings). Other typical acquisition parameters were a tube voltage of 120 kVp, a tube current of 39-464 mAs, and a rotation time < 1 s.

### Segmentation of the lesions

**Figure 1:**
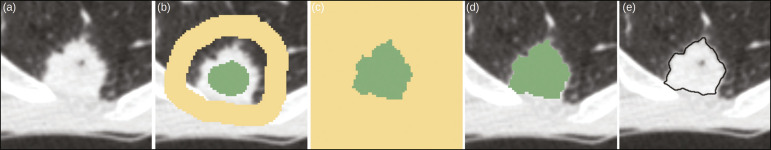
Semi-automated segmentation of a pulmonary adenocarcinoma performed with the GrowCut method. a: Lesion seen on an axial CT image with lung window settings. b: Internal and external tumor regions. c: GrowCut results. d: Removal of the external region. e: Delimitation of the lesion borders.

In order to perform the radiomic analysis, the lesions had to be segmented on the CT scans. The segmentation anatomically separates the structures or tissues seen in imaging examinations. In our study, all lesions were submitted to semi-automated segmentation with the GrowCut tool (3D Slicer, Boston, MA, USA), a interactive segmentation method^(19)^. The GrowCut method has been validated for lung cancer assessment on CT images^(20,21)^. For the semi-automated segmentation, two regions-inside and outside the tumor, respectively (Figures 1a and 1b)-were marked on three slices (axial, sagittal, and coronal) with lung window settings, at a level of −500 HU and a width of 1,400 HU. The tumor tissue was then detected in three dimensions with the GrowCut algorithm (Figure 1c), after which the external portion of the tumor was removed (Figure 1d) and the tumor borders were delimited (Figure 1e). Finally, the tumor imaging data were exported as a structured Digital Imaging and Communications in Medicine for Radiation Therapy file^(22)^ to be used in the radiomic feature extraction process.

### Radiomic features

**Figure 2:**
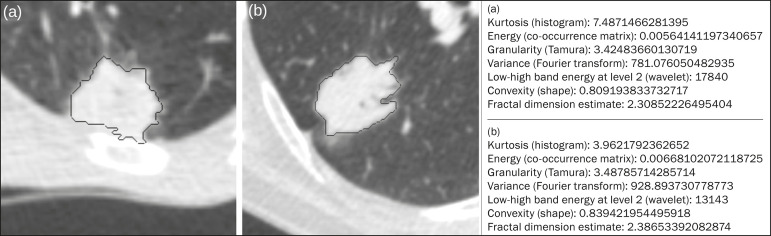
Examples of tumors on axial CT images with lung window settings and their respective radiomic feature values. a: Lung adenocarcinoma in a 68-yearold male patient who evolved to death (overall survival, 55 days). b: Lung adenocarcinoma in a 77-year-old female patient who was still alive at this writing (overall survival, 2801 days).

The radiomic feature extraction process consists of a massive calculation of numerical variables that represent the visual content of an image (Figure 2). In this study, the tumors were characterized on the basis of 2,465 quantitative variables, with specific software^(23-25)^: IBEX (University of Texas MD Anderson Cancer Center, Houston, TX, USA); LIRe-JFeatureLib (Institute for Information Technology, Klagenfurt University, Klagenfurt, Austria); and ImageJ (National Institutes of Health, Bethesda, MD, USA). The radiomic features were classified into four main groups: first order, second order, higher order, and shape^(11,26)^. First-order features (gray level and histogram) individually describe the distribution of the tumor pixel values. Second-order features (co-occurrence matrix, run length matrix, and Tamura texture) describe spatial relationships between the tumor voxels. Higher-order features (neighboring gray tone difference matrix, Laplacian-of-Gaussian filters, Gabor filters, Fourier transform, Haar wavelet, and fractal dimension estimate) describe repetitive texture patterns imposed by filters or transforms. And finally, shape features describe the borders and geometric properties of the tumor.

### Survival analysis

The prognosis was analyzed on the basis of the correlation between CT radiomic features and overall survival. We used the Kaplan-Meier method to calculate the survival times and the probability of all-cause death. Patients who were still alive or had been lost to follow-up were censored for the calculations of the overall survival probabilities. Patients were grouped according to the median radiomic feature value^(14)^. The log-rank test was used in order to determine the statistical difference between the Kaplan-Meier curves in each group so as to identify the features with the greatest prognostic potential (R survival package; R Foundation for Statistical Computing, Vienna, Austria). Values of *p* < 0.05 were considered statistically significant.

## RESULTS

Twenty-eight patients died during the study, 16 of them dying from lung cancer. Seventy-three patients were censored. The mean overall survival was 1,819.4 days (95% CI: 1,481.2-2,157.5). Of the 28 patients who died, 22 (79%) were men, with a mean age of 68.7 ± 8.8 years, and 6 (21%) were women, with a mean age of 66.8 ± 7.7 years. The following clinical stage (TNM) distribution was found in our sample: T1, T2, T3, and T4 in 10 (36%), 14 (50%), 2 (7%), and 2 (7%) of the patients, respectively; N0, N1, N2, and N3 in 11 (39%), 4 (14%), 10 (36%), and 3 (11%), respectively; and M0 and M1 in 16 (57%) and 12 (43%), respectively. The following histological types were found: adenocarcinoma, in 14 cases (50%); squamous cell carcinoma, in 8 (28%); small cell carcinoma, in 3 (10%); unspecified non-small cell lung cancer (NSCLC), in 1 (4%); poorly differentiated neuroendocrine NSCLC, in 1 (4%); and adenosquamous NSCLC, in 1 (4%).

**Figure 3:**
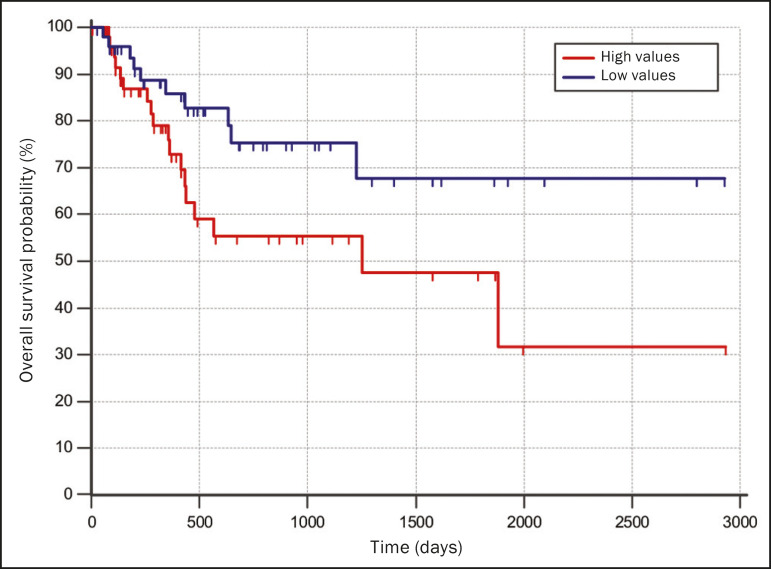
Kaplan-Meier curves of the radiomic feature mean of the Fourier transform. Each dash on the curves represents a censored patient.

The mean of the Fourier transform was the only radiomic feature that showed a statistically significant difference in the Kaplan-Meier curve analysis (Table 2). Patients with a high mean of the Fourier transform (greater than the median of 109.10) were identified as being at high risk, with a hazard ratio of 2.12 (95% CI: 1.01-4.48). That high-risk group was composed of 29 men (57%), with a mean age of 68.1 ± 9.4 years, and 22 women (43%), with a mean age of 67.1 ± 7.5 years (Figure 3). The following clinical stage distribution was seen in this group: T1, T2, T3, and T4 in 16 (31%), 25 (49%), 5 (10%), and 5 (10%) of the patients, respectively; N0, N1, N2, and N3 in 22 (43%), 10 (20%), 15 (29%), and 4 (8%), respectively; and M0 and M1 in 38 (75%) and 13 (25%), respectively. The histological types found in this group were adenocarcinoma, in 30 cases (59%); squamous cell carcinoma, in 10 (19%); small cell carcinoma, in 5 (10%); carcinoid tumor, in 2 (4%); large cell carcinoma, in 1 (2%); unspecified NSCLC, in 1 (2%); poorly differentiated neuroendocrine NSCLC, in 1 (2%); and adenosquamous NSCLC, in 1 (2%). Patients with a low mean of the Fourier transform (less than or equal to the median of 109.10) were identified as being at low risk, with a hazard ratio of 0.47 (95% CI: 0.22-0.99).

**Table 2:** Radiomic features of higher prognostic potential for lung cancer obtained from contrast-enhanced CT images in our sample (N = 101).

Radiomic feature (range)	Values	Number of deaths	Overall survival (days) Mean (95% CI)	Hazard ratio (95% CI)	*P*-value
Mean of the Fourier transform (94.92 to 122.91)	High Low	18 10	1,465.4 (985,2-1,945.6) 2,164.8 (1,745.4 - 2,5 84.1)	2.12 (1.01-4.48) -	0.048
Co-occurrence matrix prominence (35,997.37 to 3,885,382.04)	Low High	17 11	1,190.3 (877,9-1,502.7) 2,155.8 (1,756.1-2,555.5)	2.12 (0.99-4.52) -	0.051
Co-occurrence matrix correlation (-0.049 to 0.635)	Low High	17 11	1,594.8 (1,106.7-2,082.9) 2,051.5 (1,608.9-2,494.1)	2.07 (0.98-4.40) -	0.057
Co-occurrence matrix cluster shade (-37,575.62 to 4,516.65)	High Low	17 11	1,157.2 (840,3-1,474.1) 2,101.9 (1,669.8-2,534.0)	2.08 (0.98-4.43) -	0.058
Highest value of the Fourier transform (189 to 238)	High Low	18 10	1,483.4 (1,014.4-1,952.4) 2,226.3 (1,847.1-2,605.4)	2.04 (0.97-4.31) -	0.061

**Figure 4:**
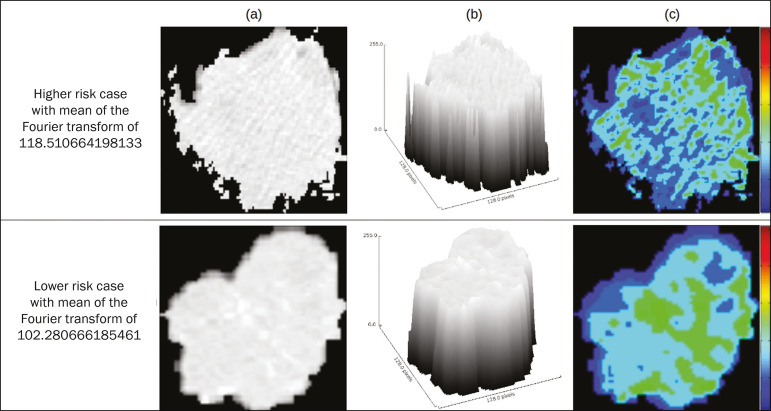
Quantification of tumor heterogeneity in the lesions, stratified by the mean of the Fourier transform. a: Axial CT image, with lung window settings, showing the segmented tumor. b: Three-dimensional distribution of the gray levels. c: Map reflecting the local energy of the tumor with a 5 × 5 pixel window.

Figure 4 illustrates the tumor heterogeneity quantification in two lesions of the risk groups stratified on the basis of the mean of the Fourier transform. In comparison with the lower-risk lesions, the higher-risk lesions showed greater heterogeneity, characterized by a larger number of peaks in the chart showing the three-dimensional distribution of the gray levels and by the presence of more infiltrating regions in the local energy map.

## DISCUSSION

Radiomics has proven to be a promising tool in the development of quantitative biomarkers for medical imaging, increasing diagnostic accuracy, improving prognostic assessment, and supporting personalized medicine^(11,17)^. In the present study, we investigated the association between quantitative radiomic features on CT images and overall survival in patients with malignant lung neoplasms. In our sample, a radiomic feature related to tumor heterogeneity (the mean of the Fourier transform) correlated with overall survival.

Studies have shown that the features used for clinical staging in the TNM system are not the only CT features of primary lung tumors that correlate with prognosis. For example, the presence of cavities and the total tumor volume (including non-solid components) are factors that correlate with prognosis in patients with squamous cell NSCLC and adenocarcinoma, respectively^(2)^. There are studies that have quantitatively assessed NSCLC using radiomics. Van Timmeren et al.^(27)^ presented three CT features with prognostic value for NSCLC: the mode (most common value) of the image histogram after the use of a Laplacian-of-Gaussian filter, the mean intensity of a volume centered on the voxel with the highest gray level, and the inverse variance of the co-occurrence matrix calculated after a wavelet transform. Carvalho et al.^(28)^ discovered the short-run gray-level emphasis of the gray-level run length matrix on positron emission tomography images combined with CT, a feature that correlates with prognosis in patients with lung neoplasms. Aerts et al.^(14)^ identified a radiomic signature associated with survival in patients with NSCLC composed of four features: (I) first-order energy, (II) shape compactness, (III) non-uniformity of the gray-level run length matrix, and (IV) non-uniformity of the gray-level run length matrix after a wavelet transform.

In the present study, we identified a radiomic signature, related to tumor heterogeneity, on CT images of lung lesions to have prognostic value for patients with lung cancer. To our knowledge, this finding has not been reported previously, except in a preliminary study presented in abstract form at a scientific conference. Radiomics-based prognostic assessment on chest CT images is an objective, noninvasive, low-cost method with great potential for use in routine clinical practice, depending only on appropriate scientific validation and definition of models for its inclusion as a tool in actual clinical settings^(29)^. Conceptually, the Fourier transform is used in order to obtain imaging features in the frequency domain. The mean frequency of the spectrum after the fast Fourier transform is associated with variations in smoother or rougher texture patterns, therefore being related to tumor heterogeneity^(29)^. Higher-risk lesions (overall mortality) had higher means of the Fourier transform and rougher, less uniform textures, whereas lower-risk lesions had lower means of the Fourier transform and smoother, more uniform textures.

Our study has some limitations. First, the sample size was relatively small. We chose to study only contrast-enhanced CT scans of diagnostic quality that were acquired by following an appropriate clinical protocol, rejecting unenhanced images and those with significant artifacts. Although all examinations included in our study followed the same clinical protocol for contrast medium administration, no corrections were made for possible differences in volume and flow rate depending on patient body type or cardiac status, factors that can affect enhancement patterns. In addition, we included examinations performed on CT scanners with and without iterative reconstruction, which can also influence image resolution and the features analyzed. The number of deaths to be used in the overall survival analysis was also limited; only 16 deaths were directly related to lung cancer, which decreased the statistical power of our survival analysis. That is probably due to the fact that we focused on smaller (mainly T1 and T2) lesions, having a multidisciplinary discussion about the best course of action, whereas more aggressive tumors, which are associated with worse prognoses and shorter survival times, were excluded. Furthermore, the various courses of action dependent on the different types of lung neoplasms and the medications available can also affect the survival analysis and the prognosis. Studies like the present one serve as a proof of concept, showing the applicability of the radiomic model as a prognostic tool. However, for the effective inclusion of radiomics in the clinical setting, further studies should be conducted and models for its use within the diagnostic imaging workflow should be established. For example, it is important and advisable to conduct stability and reproducibility analyses of radiomic variables in larger volumes of data, in order to gather more evidence about the robustness of the features and to validate the radiomic approach. Although radiomics is meant to be more comprehensive and include aspects related to prognostic assessment and to therapeutic decision-making, it has conceptual and methodological bases in common with computer-aided diagnosis. The U.S. Food and Drug Administration has recently defined a set of rules aimed at facilitating the approval process of computer-aided detection systems^(30)^. These rules stipulate, among other requirements, that the documentation of the products include a detailed description of the patient population for which the system is indicated and a detailed description of the compatible equipment and compatible image acquisition protocols, as well as possible warnings and discussions about the product limitations, including situations in which the device can fail or may not achieve the expected performance level (e.g., because of poor image quality or use with certain subpopulations), as applicable. These rules aim to prevent, or at least minimize, performance variations in the process of image pattern recognition, enabling a more widespread use of the computer solution, assuming that the boundary conditions have been guaranteed. It is expected that something similar will be defined for the computational algorithms used in radiomics.

In conclusion, the present study investigated whether different radiomic methods could be considered effective quantitative biomarkers in images of malignant lung neoplasms. We identified a radiomic CT signature based on the Fourier transform that is potentially useful for prognostic assessment, risk stratification, and quantification of tumor heterogeneity in patients with lung cancer.
